# Use of surrogate endpoints in health technology assessment and reimbursement of treatments for the management of chronic kidney disease

**DOI:** 10.1016/j.eclinm.2025.103465

**Published:** 2025-08-29

**Authors:** Rod S. Taylor, Hiddo J.L. Heerspink, Marc Buyse, Oriana Ciani, Bruno Detournay, Daniel Gallego, Juan Carlos Julián Mauro, Smeeta Sinha, Marta Trapero-Bertran, Lesley A. Inker

**Affiliations:** a Robertson Centre for Biostatistics & General Practice and Primary Care, School of Health and Wellbeing, University of Glasgow, Glasgow, UK; b Department Clinical Pharmacy and Pharmacology, University of Groningen, University Medical Center Groningen, Groningen, the Netherlands; c International Drug Development Institute (IDD), Biostatistics, Louvain la Neuve and University of Hasselt, I-BioStat, Hasselt, Belgium; d Center for Research on Health and Social Care Management (CERGAS), SDA Bocconi, Milan, Italy; e CEMKA, Bourg-la-Reine, France; f European Kidney Patients Federation, Federación Nacional ALCER, Madrid, Spain; g European Kidney Patients Federation, Nacional de Asociaciones ALCER & School of Psychology, Universidad Autónoma de Madrid, Madrid, Spain; h Donal O’Donoghue Renal Research Centre, Northern Care Alliance NHS Foundation Trust, Salford & Manchester Academic Health Sciences Centre, University of Manchester, Manchester, UK; i Department of Economics and Business, Faculty of Law, Economics and Tourism, Universitat de Lleida, Lleida, Spain; j Tufts Medical Centre Department of Medicine, Boston, USA

**Keywords:** Surrogate outcomes, Health technology assessment, Reimbursement, Chronic kidney disease

## Abstract

The judicious use of surrogate endpoints as substitutes for patient relevant target outcomes can substantially reduce the size and duration of clinical trials, thereby driving down research and development costs and driving faster patient access to innovative therapies. Whilst increasingly used by regulators over the last two decades, health technology assessment (HTA) agencies and payers have been more sceptical in acceptance of surrogates in their reimbursement decisions. Central to acceptance is scientific validation and demonstration of the association in the treatment effect on the surrogate endpoint and target outcome. This review summarises the validity and utility of glomerular filtration rate (GFR) slope as a ‘first in class’ surrogate with robust evidence of a strong treatment effect association (i.e., R^2^ trial of 97%) with clinically meaningful kidney target outcomes including dialysis and kidney transplantation. Given the likely continued challenges in the use of surrogate endpoints in future healthcare policy making, we conclude this review with the opportunities for stakeholders–healthcare industry, regulators and payers, clinicians and trialists, and patients and the public–to leverage the future appropriate use of surrogates.

**Funding:**

None declared.

**Search strategy and selection criteria**

Data for this review were based on a previous scoping literature review of the use of surrogate endpoints in clinical trials and health policy that undertook searches of a number of bibliographic databases (EMBASE, MEDLINE, Cochrane Methodology Register) using search terms including “surrogate end points”, “guidelines”, and “trials” for articles published in English from inception to May 2022.^1^ Searches were updated (January 2025) in combination with the term “chronic kidney disease” and supplemented with targeted searches of national health technology assessment agency websites and the references of key relevant articles known to the authors.

## Introduction

Chronic kidney disease (CKD) presents a significant burden to global healthcare systems. CKD affects approximately 10–15% of the global adult population, with an increasing prevalence due to factors such as diabetes, hypertension, obesity and aging populations.^2^ CKD is projected to become the fifth leading cause of death worldwide by 2040.^3^ The costs of treatment, including dialysis, kidney transplantation, and lost productivity are substantial with an estimated worldwide CKD-related cost in 2019 at over US$1 trillion.^4^ Timely access to evidence-based treatments is crucial for alleviating the future burden of CKD.

Access to healthcare treatments should ideally be based on appropriately designed, conducted, analysed and reported randomised controlled trials (RCTs) that directly measure patient relevant final target outcomes, such as overall survival, severe morbidity, or health-related quality of life. However, surrogate endpoints are increasingly used as trial primary endpoints to accelerate regulatory approval and patient access to new therapies by substituting for direct target outcomes.^5^ With faster accrual, surrogate endpoints can offer the advantages to patients, clinicians, and researchers of reducing the duration, size, and total cost of trials for chronic diseases like CKD where the definitive impact of a new treatment on a target outcome can be difficult to assess due to the slow progression of the disease.^6^

Kidney failure requiring replacement therapy (i.e., dialysis or a kidney transplant) is a serious but relatively rare consequence of CKD that occurs after many years in most patients.^7^ Glomerular filtration rate (GFR) slope, a biomarker reflecting of changes in kidney function over time, has gained recent attention as a validated surrogate endpoint for CKD therapies with very strong evidence of its prediction of long-term patient-relevant kidney failure outcomes.^8^ The United States Food and Drug Agency (FDA) and the European Medicines Agency (EMA) have recently approved GFR slope as an acceptable primary endpoint for trials of CKD therapies.^9^

Beyond licensing approval, most advanced healthcare systems impose an additional regulatory requirement–health technology assessment (HTA)–which must be met before therapies are funded. HTA seeks to assess the broader health value of new therapies, informing healthcare payers whether the therapy should be reimbursed, be that through a public funding or a social insurance system.^10^ Alongside the licensing requirements of efficacy and safety, HTA agencies typically require the additional evidentiary criteria of comparative effectiveness (i.e., does the therapy provide sufficient health benefit over the longer term compared to standard of care?) and incremental cost-effectiveness (i.e., does this benefit relative to the cost of therapy represent good value for money?).^10^

Whilst surrogate endpoints can play a key role in both the licensing and HTA/reimbursement settings, in contrast to regulators such as the FDA and EMA, HTA agencies and payers have traditionally been more cautious in their access decisions around new treatments with clinical trials based on surrogate endpoints rather than target outcomes.^2^

Reliance on surrogate endpoints may result in inaccurate value assessment due to systematic overestimation of clinical benefit and cost-effectiveness, leading to new treatments initially gaining market access but later being either rejected or granted only limited reimbursement and coverage by HTA bodies.^2^ This highlights the need for appropriate methods for surrogate validation to minimise uncertainty related to their use.

Given the wide diffusion of surrogates as primary endpoints in clinical trials, this position paper seeks to provide scientific and policy direction for the appropriate use of surrogate endpoints to inform funding decisions and timely access for new treatments in the management of CKD. The paper is organised into four subsections. First, we present a framework for the definition and trial-based evaluation of surrogate endpoints, with emphasis on the importance of statistical validation. Second, we discuss how HTA agencies currently approach the use of surrogates in their reimbursement decision-making with illustration related to recent new treatments for CKD. Third, we provide an overview of the validation evidence that supports GFR slope as a surrogate for kidney failure in the management of CKD. Finally, we offer recommendations for the future use of surrogate endpoints to inform HTA evaluation and access to future new CKD treatments.

## Validation of surrogate endpoints

Based on concepts developed in the literature over the last two decades,^10^^,^^11^ ‘the Ciani framework’ for surrogate endpoints has become widely accepted by the international HTA community.^12^, ^13^, ^14^, ^15^ This framework proposes three levels of evidence in the evaluation or ‘validation’ of a surrogate endpoint (see Table 1)–biological plausibility for an outcome to be a good surrogate endpoint for clinical benefit (level 3)–association between the surrogate endpoint and the target outcome at the individual level based on observational evidence or trial data (level 2)–‘individual level surrogacy’; and association between the treatment effect of the surrogate and the target outcome based on RCT data (level 1)–‘trial level surrogacy’. Level 1 evidence or trial level surrogacy is considered the most important in HTA decision-making. Demonstration of level 1 evidence is typically based on meta-analytic methods and requires data from RCTs that have assessed both the surrogate endpoint and target outcome.

**Table 1:** HTA framework for evaluation of surrogate endpoints.

	Definition	Source of evidence	Statistical metrics
Level 3	Biological plausibility	Clinical data and understanding of disease (surrogate endpoint lies on the disease pathway with final patient relevant outcome)	Not applicable
Level 2	Observational association	Epidemiological studies and/or clinical trials demonstrating the relationship between surrogate endpoint and target patient relevant outcome	Correlation between surrogate endpoint and target patient relevant outcome
Level 1	Interventional/treatment effect association	Randomised controlled trial(s)^a^ demonstrating association between the treatment change in surrogate endpoint and target patient relevant outcome^b^	Trial level R^2^ Spearman’s correlation Surrogate threshold effect (STE)

a Individual participant or trial level meta-analysis of multiple randomised controlled trial or single large randomised controlled trial with surrogate-final outcome association assessed by trial site.

b Evidence should be randomised controlled trials from same disease indication, line of therapy, class of treatment/intervention and comparator therapy that surrogate endpoint is applied. If extrapolating from different disease indication, line of therapy, class of treatment/intervention and comparator therapy then evidence may not qualify as level 1.

The strength of the association based on the trial level effects of treatment on the surrogate and target outcome is quantified using metrics including the coefficient of determination (R^2^ trial), Spearman’s correlation coefficient (ρ), or Kendall’s tau.^12^^,^^16^ Individual participant/patient data (IPD) meta-analysis remains the optimal approach to surrogacy evaluation as it enables the standardisation of statistical methods across IPD sets and robust analysis at both the patient and trial levels, i.e., allowing assessment of level 1 and 2 evidence of surrogacy. However, as IPD is often not available, with the appropriate statistical methodology, surrogate validation can be based on meta-analyses of published aggregate trial level data. Statistical extensions to meta-analysis have been developed to ensure robust evaluation of surrogate endpoints that include multivariate and Bayesian methods.^17^^,^^18^ Surrogate endpoint evaluation using meta-analysis be conducted according to the recent ‘reporting of surrogate endpoint evaluation using meta-analyses’ (ReSEEM) guidelines.^19^

**Fig. 1:**
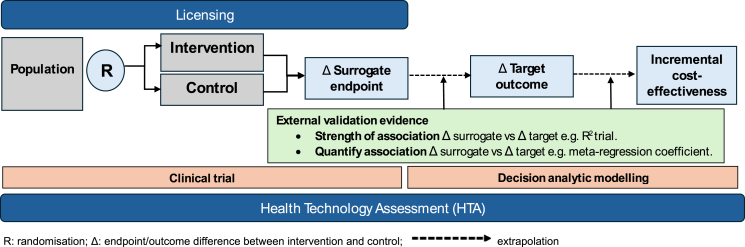
**Schematic framework of the use of surrogate endpoints in licensing and HTA**.

In addition to evidence of a strong statistical association between the surrogate endpoint and patient relevant outcome, the HTA process may also require quantification of the predictive effect on change on the target outcome to enable estimation of a therapy’s cost-effectiveness (e.g., cost per quality adjusted life year (QALY)) based on the observed effect of the treatment on the surrogate. An increasingly reported metric is the surrogate threshold effect (STE), which is the magnitude of treatment effect on the surrogate that would predict a significant treatment effect on the target outcome.^20^ A schematic of the process for handling surrogate endpoints in licensing and HTA is shown in Fig. 1 and example of this process shown in Supplementary eFig. 1.

Caution is needed in extrapolating the evidence surrogate endpoint validation. For example, low density lipoprotein (LDL)-cholesterol has been shown to be a valid surrogate endpoint for cardiovascular related mortality statins it appears to be less predictive for other classes of lipid lowering therapies such as fibrates.^21^ Surrogate validation should therefore be based on RCTs with the appropriate range of populations, interventions, comparators and outcomes (“PICO”) reflective of the specific HTA decision problem.

## Current policy and practice of HTA agencies on use of surrogate endpoints

The use of surrogates was originally conceived in the limited context of specific drug approval circumstances (e.g., US FDA’s accelerated approval programme) to enable earlier licensing for therapies to treat serious conditions and addressing a major unmet medical need. One of the earliest examples was the acceptance of RCT evidence showing increases in CD4 counts (surrogate endpoint) for improvements in overall survival resulting in approval of the first wave of antiviral drug treatments for acquired immunodeficiency syndrome (AIDS). Since that time, the use of surrogate endpoints in drug approval has grown considerably. In 2018, the US FDA publicly released an ‘Adult Surrogate Endpoint Table’ of more than 100 surrogate endpoints that may be used as primary endpoints in clinical trials that form the basis of traditional or accelerated approval of new drugs or biologics and are “reasonably likely to predict clinical benefit”.^22^ Whilst, in principle, a surrogate endpoint must be fully validated to be used in lieu of the target outcome for full approval, recent reviews have shown that over 50% of all drug and biologic approvals by FDA and EMA are based on surrogate endpoints.^5^^,^^23^

The FDA surrogate table currently lists GFR and serum creatinine for traditional approvals for treatments targeting “CKD secondary to multiple etiologies”. In September 2023, EMA published their opinion that GFR slope can be “used as a validated surrogate endpoint for CKD progression in RCTs for standard marketing authorization and indication extension approvals”.^24^ Other endpoints in CKD, including early change in albuminuria have also been proposed but are not currently approved as valid surrogates by either FDA or EMA.^9^

Whilst regulatory bodies are primarily concerned with weighing the balance of efficacy versus safety of new therapy, HTA agencies (or ‘payers’) seek to assess the wider value of treatments to the healthcare system to inform their decision to fund access/coverage. HTA value typically focuses on considerations of comparative effectiveness (clinical benefit of a therapy relative to current standard of care) and the economic consequences of the introduction of a new treatment, alongside other factors such as equity, severity of disease, or unmet need. Metrics of ‘value’ can vary considerably across different HTA agencies and countries from the formal assessment of cost effectiveness (e.g., incremental cost per QALY) to more clinical assessment of benefit (e.g., ‘Service Médical Rendu’, SMR) that are in turn used to negotiate the acceptance of a new drug/intervention and its costs.^25^^,^^26^

An analysis across nine leading HTA agencies published in 2021 found that of 291 HTA reports, 61 relied on surrogate endpoints.^11^ The handling of surrogate endpoint evidence was found to vary greatly across HTA agencies, with inconsistent consideration of the level of evidence and statistical validation. As a result, the authors called for international harmonisation in the evaluation of surrogate endpoints across HTA agencies.^11^ Based on these recommendations, an international consortia (UK National Institute for Health and Care Excellence (NICE), Canadian Drug Agency, US Institute for Clinical and Economic Review and Rubix Health, Australian Department of Health and Aged Care, Colombian Institute for Technology Assessment in Health, and Dutch National Health Care Institute (ZIN)) recently came together and published a report in early 2025 offering guidance on best practice for using surrogate endpoints in health economic models to guide HTA decisions.^27^ The 3-level HTA framework described above is central to this guidance.

As part of this review, a contemporary analysis of the use of surrogate endpoints by selected HTA agencies in France, Belgium, Germany, Italy, Spain, and UK was undertaken (see Supplementary eTable 1). It was found that there was a broad consistency, irrespective of country, that HTA agencies prefer the use of trial evidence based on target outcomes. Furthermore, surrogate endpoints should only be considered when target outcome data are not available, or their evidence is limited (e.g., immature clinical trial data on overall survival). NICE (UK), Spanish Agency of Medicines and Medical Devices (AEMPS), German Institute for Quality and Efficiency in Health Care (IQWiG), and French Authority for Health (HAS) all state that their grounds for acceptance of surrogate endpoints depend on a requirement of scientific validation. However, consistent with previous analyses, there was considerable variation in practice with only two agencies providing specification of their scientific requirements for surrogate validation. G-BA/IQWiG (Germany) will consider surrogate endpoints if they have been validated with appropriate statistical methods (as detailed in the previous section).^28^ If the surrogate endpoint is neither validated nor accepted by the consideration of a STE, the results can be presented but are not regarded as proof of added benefit by the G-BA. Similarly, NICE’s Decision Support Unit provide methodological guidance on the process for validating a surrogate endpoint the meta-analytic approach to surrogate endpoint evaluation and data requirements.^17^ Importantly, there is no current international consensus across the HTA community on the minimum threshold level for the establishment of a ‘sufficient’ association between the treatment effect on a surrogate endpoint and target outcome. However, IQWiG and the EuNetHTA guidance on outcomes^29^, ^30^, ^28^ (one of the methods documents underpinning the new EU HTA Regulation for a single joint clinical assessment (JCA) process across European Commission countries) state a correlation of ≥0.85 (equivalent to an R^2^ trial of ≥0.72) to represent a “high” association and to consider the precision in the predictions of the target outcome in their acceptance of a surrogate.

## Evidence base for the validity of surrogate endpoints in CKD

Historically, drug development for CKD has focused on patients with later stages of disease as they are at high risk of CKD progression. This is in part because the commonly used patient-relevant final or ‘target’ outcomes in clinical trials occur only after a prolonged disease course that may extend 10–20 years,^2^^,^^7^ a timeframe that is not feasible for trials of early CKD. Implementing treatments in the early stages of CKD could have a greater impact on delaying the time to progression to kidney failure and reducing the risk of progression than interventions applied at later stages of disease. As discussed above, surrogate endpoints can be used earlier in the CKD process and with smaller sample size and shorter follow-up duration.

Kidney Disease Improving Global Outcomes (KDIGO) define CKD as an abnormality of kidney structure or function, present for a minimum of 3 months with implications for health.^31^ This can include an estimated GFR < 60 ml/min per 1.73 m^2^ or other marker of kidney damage, which is most commonly, elevated albuminuria. Thus, GFR and urine albuminuria have been extensively evaluated as candidate surrogate endpoints because they are the most widely used biomarkers in CKD.^8^^,^^9^ The urine albumin concentration is often divided by the urine creatinine concentration to account for variations in urine dilution between individuals. This is a central reason for the recommendation to use it measured as urine albumin to creatinine ratio in a spot urine, to detect and stage CKD. In specific disease, total protein or urine albumin assessed using 24-h urine collections are also used. Here on in, we will continue to use the general term urine albumin (or albuminuria) to refer to all of the variations.

Over the past two decades, the Chronic Kidney Disease Epidemiology Collaboration (CKD-EPI) have led the evaluation of the validity of changes in GFR and albuminuria as surrogate endpoints for clinical trials in CKD.^8^^,^^9^ Level 3 evidence (biological plausibility) for GFR decline as an endpoint of CKD progression is very strong. Change in GFR over time is the definition for the progression and remission of kidney disease–patients cannot reach kidney failure in the absence of a decline in GFR. Albuminuria is generally caused by increased permeability of the glomerular capillary wall to macromolecules. Experimental studies showed that increased tubular exposure to albumin promotes activation of inflammatory mediators causing tubulo-interstitial injury and further kidney damage. Albuminuria would not be expected to be a surrogate across all CKD etiologies. Unlike GFR, its use would require understanding that the specific disease affects albuminuria and that the intervention is expected to impact its change.

For GFR, there is strong scientific evidence to support surrogacy at both level 1 and level 2. CKD-EPI has provided evidence for validation of surrogate endpoints using meta-analyses of IPD to evaluate both the epidemiological associations between changes in GFR to subsequent development of end stage kidney disease requiring kidney replacement therapy (KRT), and trial level analyses examining the association of treatment effects on the surrogate endpoints to kidney failure, using Bayesian mixed-effects meta-regression analyses.^8^^,^^32^^,^^33^

**Fig. 2:**
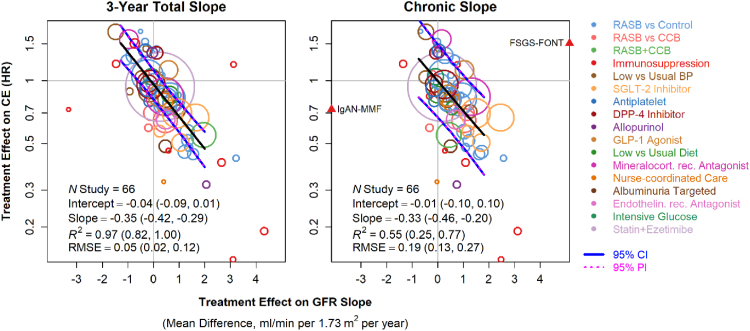
**Trial level analyses for the association between treatment effects on GFR slope and target outcome of kidney failure with replacement therapy.** Shown in each plot is the relationship between estimated treatment effects on the target outcome (kidney failure with replacement therapy (KFRT), GFR < 15 mL/min per 1.73 m^2^ or doubling of serum creatinine), as indicated on the vertical axis, with the estimated treatment effects on the GFR slope, as indicated on the horizontal axis. Treatment effects on GFR slope are expressed as mean differences for the treatment minus control groups and are expressed in mL/min per 1.73 m^2^/year. Treatment effects on the clinical endpoint are expressed as hazard ratios. Each circle is a separate intervention with the size of the circle proportional to the number of events. The colors of each circle indicate intervention type. The black line is the line of regression through the studies. The blue lines are the 95% pointwise Bayesian confidence band. The associations between the treatment effects were modeled using Bayesian meta-regression within R using the RStan package.^36^ The pink dashed lines are the 95% pointwise Bayesian prediction bands computed from the model. RASB, renin angiotensin system blockers; CCB, calcium channel blocker; BP, blood pressure; DPP-4, Dipeptidyl peptidase 4; GLP-1, Glucagon-like peptide-1, RMSE, Root mean square error; SGLT2, Sodium glucose cotransporter.

In meta-analyses of IPD from 66 RCTs, treatment effects on the mean changes in GFR and time to thresholds of GFR decline of 30–40% are strongly associated with treatment effects on the clinical endpoint, most commonly defined by the composite outcome of KRT, doubling of serum creatinine or GFR < 15 ml/min per 1.73 m^2^.^8^^,^^33^ Many interventions in GFR cause an immediate change in GFR that is different from the longer-term chronic phase.^34^ Thus, changes in GFR trajectories can be expressed as total change from baseline or change in the chronic phase.^35^ There was strong agreement between the treatment effects on the 3-year total slope and on the clinical endpoint with an R^2^ trial of 0.97 (95% Bayesian credible interval (BCI): 0.82–1.00) (Fig. 2 and Supplementary eTable 2).^8^^,^^34^ The slope of the meta-regression line is −0.35 (95% BCI: −0.42 to −0.29) per mL/min per 1.73 m^2^/year), indicating that a 0.75 ml/min per 1.73 m^2^/year greater beneficial effect of the treatment on the total GFR slope is associated with an average 23.0% lower hazard ratio for the clinical end point (95% BCI 20.0%–27.0%). The intercept of the meta-regression is −0.04 (95% BCI: −0.09 to 0.01), indicating that in the absence of a treatment effect on the 3-year total slope, the average treatment effect on the clinical endpoint is likely to be small (i.e., 95% probability for the hazard ratio to be between 0.91 and 1.01). The strength of this trial-level association indicates a uniquely strong surrogate endpoint and is considerably stronger than other surrogate endpoints that are regularly used in other fields, such as oncology (e.g., progression free survival for overall survival) and cardiovascular disease (e.g., systolic blood pressure for stroke and cardiovascular mortality). Furthermore, this association was consistent across disease and by severity of CKD as defined by baseline level of GFR, albuminuria and rate of progression in the study’s control arm.^8^^,^^34^^,^^35^^,^^37^

For the chronic GFR slope, analysis showed an R^2^ trial of 0.55 (95% BCI: 0.25–0.77) (Fig. 2). The slope of the meta-regression line differs substantially from 0, at −0.33 (95% BCI: −0.46 to −0.20) indicating that a 0.75 ml/min per 1.73 m^2^ per year greater beneficial treatment effect on the total GFR slope is associated with an average 21.9% lower hazard for the clinical end point (95% BCI: 13.9%–29.2%). That the intercept of the regression line is nearly 0.00 (−0.01, 95% BCI −0.10 to 0.10) indicates low risk of a false negative conclusion of the absence of a treatment effect on the clinical endpoint when there is no treatment effect on chronic slope (i.e., 95% probability for hazard ratio to be between 0.90 and 1.11).

Results were also consistent for a stricter definition of the target outcome as KRT or GFR < 15 ml/min per 1.73 m^2^ (Table 1). For the total slope, compared to the analysis of the primary clinical endpoint the median R^2^ decreased from 0.97 (0.82, 1.00) to 0.92 (0.56, 0.99). For the chronic slope, the median R^2^ increased from 0.55 (0.25, 0.77) to 0.87 (0.64, 0.97), although BCIs for the secondary clinical endpoint were wider than for the clinical endpoint, indicating reduced precision.

For albuminuria, like GFR, both higher levels of baseline and increases in albuminuria were also associated with faster progression of CKD.^38^ In trial level analyses using IPD of participants with baseline albumin to creatinine levels of 30 mg/g or higher, treatment effects on mean change in albuminuria over 6 and 12 months had moderately strong associations with treatment effects on KRT with R^2^ of 0.69 (95% CI: 0.08–0.98).^39^ The trial level associations were consistent across subgroups of disease cause, including CKD not otherwise stated, diabetes or IgA nephropathy.^40^

These scientifically validated GFR based endpoints have been accepted by regulatory agencies including qualification of GFR decline as a surrogate endpoint by EMA applied in numerous RCTs. They are being used in numerous trials, including as an endpoint to support full approval.^24^^,^^40^ In IgA nephropathy, changes in albuminuria are being used as an endpoint for conditional approval along the accelerated approval pathway with GFR slope used as the confirmatory endpoint.^40^ Changes in albuminuria are also being used in phase 2 and dose-finding studies to demonstrate proof of concept in prevalent CKD, as a bridging biomarker to translate the obtained efficacy evidence of a therapy from one population to another and is currently under review as full approval endpoint in focal segmental glomerulosclerosis.^41^

Together, these data provide strong level 1 evidence to support the use of change in GFR in RCTs for evaluating of therapies aimed at improving CKD progression. However, GFR slope might not be appropriate for all studies. Selection of the right endpoint for a future study requires consideration of the unique aspects of the clinical trial including phase, specific population, treatment, and design. Indeed, even the clinical endpoint could result in false conclusions about treatment benefit if applied in the wrong settings, such as too short a trial or in a population that is not expected to reach the endpoint over the duration of the trial but nevertheless has progressive CKD.^7^ Albuminuria can similarly provide a validated surrogate, but the advantage over GFR slope is generally seen for very short studies.^42^ Additionally, the use of GFR slope and albuminuria allows for the inclusion of all participants in a study, including those who may not progress to the clinical endpoint within the time-period of the trial.

## Recent HTA assessment of new CKD treatments

To illustrate how HTA agencies approach their evidence review for new treatments for CKD, we considered the case study of sodium-glucose cotransporter-2 (SGLT-2) inhibitors—empagliflozin and dapagliflozin (see Supplementary eTable 3). Regulatory approval of both drugs are based on the RCTs of EMPA-KIDNEY^36^ and DAPA-CKD^39^ both with a primary endpoint of progression of kidney disease (defined as end-stage kidney disease i.e., a sustained decrease in eGFR to <10 ml per minute per 1.73 m^2^, a sustained decrease in eGFR of ≥40% from baseline, or death from renal causes) or death from cardiovascular causes or sustained decline in the estimated GFR of ≥50%, end-stage kidney disease, or death from renal or cardiovascular causes respectively.

As discussed above, a key element of HTA evaluation is demonstration of cost-effectiveness. Decision analytic models have been published for both empagliflozin and dapagliflozin showing their cost-effectiveness in addition to current standard therapy.^43^^,^^44^ Based on the outcome findings from the DAPA-CKD trial, McEwan et al. showed dapagliflozin compared to placebo and standard of care had incremental cost-effectiveness ratios of US$8280, US$17,623, and US$11,687 per QALY in the UK, Germany, and Spain, respectively, indicating cost-effectiveness at their country willingness-to-pay thresholds (i.e., UK: US$27,510 per QALY; Germany and Spain: US$35,503 per QALY).^43^ Over a 50-year time horizon in the UK setting, the analyses of Ramos et al. reported that empagliflozin plus standard care resulted in an incremental gain in life years (+1.04) and QALYs (+0.84), while decreasing per-patient costs by £6,019, well below the willingness to pay (WTP)’ threshold used in the UK of £20,000/QALY.^44^

Between 2021 and 2024, UK HTA bodies (NICE and Scottish Medicines Consortium (SMC)), HAS, IQwiG/G-BA, RedATS/AEMPS, and AIFA evaluated SGLT-2 inhibitors for CKD. Their approval recommendation and their consideration of trial evidence base are detailed in Supplementary eTable 3. Empagliflozin and dapagliflozin were recommended for funding by all agencies. Interestingly, agencies noted that whilst the treatment effect on a composite/multiple primary endpoint was predominated by a decline in GFR, the drug effects of the clinical components of the clinical outcome were also supportive.

Whilst this case study is limited to only SGLT-2 inhibitors for CKD and the formal assessment by only a small number of HTA agencies, it does indicate when part of a multiple outcome including CVD events and renal mortality, improvement in the endpoint of GFR decline can be acceptable to support coverage decisions for new treatments for CKD.

## Future challenges and opportunities

In this paper we have presented: (1) that scientifically validated surrogate endpoints can substantially improve the feasibility and costs of clinical trials with shorter follow up in smaller populations, (2) surrogate endpoints offer ethical benefits of potentially reduced risk of harms in trials to participants and faster access to beneficial new therapies in broader populations, (3) GFR slope is a ‘first in class’ surrogate endpoint with robust data supporting of very strong treatment effect association with final outcomes in CKD; and (4) clinical trial data demonstrating superiority on GFR slope has underpinned the reimbursement of SGLT-2 inhibitors for CKD by a number of international HTA agencies.

However, we also highlight that the acceptance of surrogate endpoints in CKD (and other clinical and public health areas) in the approval and reimbursement of future therapies pose challenges, especially for HTA. We therefore close this paper by considering the opportunities for stakeholders–healthcare industry, regulators and payers, clinicians and trialists, and patients and the public–to address these challenges and thereby leverage the appropriate use of surrogate endpoints in future healthcare policy making (see Table 2).

**Table 2:** Future use of surrogate endpoints in trials and decision making: challenges and opportunities.

Stakeholder	Challenges	Opportunities
Healthcare industry	- Regulatory uncertainty around acceptance of surrogate endpoints - Risk of late-stage trial failure if surrogate does not correlate with hard outcomes	- Seek early scientific advice with regulators/HTA to align trial design including the proposed use of surrogate endpoints - Invest early in parallel process of evidence generation to demonstrate surrogate endpoint validity
Regulators & HTA	- Ensuring surrogate endpoints are clinically meaningful and predictive of long-term benefit - Variability in current international HTA standards	- Provide transparent guidance for consideration of surrogate endpoints - Promote harmonisation of international standards across HTA agencies - Support models of approval and reimbursement to facilitate collection of confirmatory evidence on long-term target outcomes
Clinicians & trialists	- Whilst clinicians rely on surrogates in their practice, concern over clinical relevance of trials using surrogate endpoints - Need for consensus on standard endpoints - Lack of familiarity with regulatory requirements	- Involve of clinical professional communities in validation of surrogate endpoints - Better reporting of clinical trials using surrogate endpoints - Promote inclusion of validated surrogate endpoints in core outcome sets - Provision of education around appropriate use of surrogates
Patients & the Public	- Lack of understanding of what surrogate endpoints mean - Risk of being misled about treatment benefits - Mistrust if endpoints don’t translate to real-world benefit	- Improve communication about the meaning and limitations of surrogate endpoints - Involve patient advocacy groups in trial design - Ensure transparency about evidence linking surrogate to target outcome

### Healthcare industry

The incorporation of surrogate endpoints in clinical trials provide a prime opportunity for the healthcare industry to mitigate their rising tide of R&D costs. However, industry face the risks of regulatory acceptance of surrogates and late-stage trial failure of a surrogate that does not correlate with target outcomes. Nevertheless, early scientific advice and parallel evidence development are two key opportunities for industry to mitigate the risks of using surrogate endpoints. Early scientific advice is a formal process through which the healthcare manufacturers can consult with regulators to seek feedback on planned evidence generation strategies.^45^ Pay for service models of early advice available include National HTA scientific advice (e.g., NICE in the UK, G-BA in Germany, HAS in France) or parallel consultations with regulators and HTAs, joint EMA–HTA advice. Furthermore, the new EU HTA regulation enacted in January 2025, for a joint clinical assessment process, offers an opportunity for advice across HTA agencies of member states.^28^ Although non-binding, early advice provides industry the opportunity check their clinical trial PICO, and where relevant, and decide whether the choice of surrogate primary endpoints is appropriate from a regulatory/reimbursement perspective. Second, alongside the design and conduct of their pivotal clinical trial(s), industry need to initiate a parallel process of evidence development to underpin the scientific validity of the surrogate endpoint (see above) so that this can be incorporated into their submission dossier with regulators and HTA.

### Regulators and HTA

Whilst the FDA and EMA have a long tradition of approving pharmaceuticals and biologics based on surrogate endpoints, HTA agencies and payers have been more cautious in their acceptance. The case study of GFR slope in CKD shows that it is possible to achieve levels of scientific validation that can substantially mitigate the uncertainty of HTA agencies in their acceptance of a surrogate endpoint in their reimbursement decision. A future more balanced consideration of surrogate endpoints requires greater transparency as to the scientific requirements of individual HTA agencies as well as the establishment of international HTA standards for consideration of surrogate endpoints. Although challenging, such standards need to address the minimum strength of association (and precision of this association) between the treatment effects measured using surrogate and final outcomes necessary to establish surrogacy.^14^^,^^15^ As outlined earlier, validation of a surrogate end point is rather a ‘chicken-and-egg problem’, in that it requires evidence of the relation between the surrogate and the target outcome. However, as the target outcome for a specific new treatment will not yet be available–and hence the surrogate is used as replacement—previous trials of treatment in same class are often used to provide that evidence. Moving forward, there is a unique opportunity for regulators and HTA to link their licensing and reimbursement decisions, based on surrogate endpoints to a conditional decision, that incentivises extension of follow-up to accrue the relevant evidence on target outcomes to inform a later and more definitive decision.^15^

### Clinicians and trialists

Clinicians can be concerned with the relevance of surrogate endpoints to their individual patient decisions because a treatment may improve a surrogate without improving, or even worsening, the target patient relevant outcome(s). Educational strategies are therefore needed to improve the knowledge and understanding of clinicians on appropriate use of surrogates to their medical practice. The Journal American Medical Association (JAMA) ‘Users Guide to the Medical Literature’ online textbook chapter on surrogate endpoints is excellent example of such an educational resource.^46^ Clinical professional and clinical trial communities need to collaborate on the future appropriate selection and validation of surrogate endpoints. An exemplar is the CKD-Epidemiology Clinical Trials (CKD-EPI-CT) consortium that spun out of CKD-EPI initiative established in 2003 ‘to address fundamental questions in CKD epidemiology using individual patient data and rigorous methods in clinical chemistry and statistics to create useful tools for research, patient care, and public health’. Supported by funding from National Kidney Foundation and industry sponsors, CKD-EPI-CT coordinated the trial level meta-analysis of the statistical validation of GFR slope and its subsequent publication.^8^

There have been calls to improve the standards of reporting RCTs that use a primary surrogate endpoint.^47^ A review of trials published in high impact general medica journals in 2005 and 2006 found that 17% (107/626) used a surrogate primary endpoint but only a third discussed whether the surrogate was validated.^48^ The recently published SPIRIT-Surrogate and CONSORT-Surrogate extensions of the Recommendations for Interventional Trials (SPIRIT) and the Consolidated Standards of Reporting Trials (CONSORT) provide checklists for the reporting protocols and trial reports for trials using a surrogate endpoint as a primary outcome.^49^^,^^50^ Clinical trial groups are increasingly developing core outcome sets within their specific clinical areas to provide standardisation and harmonisation of outcome reporting in trials by identifying outcomes perceived to be fundamental for decision making within a specific area.^51^ To inform future trial design and their appropriate selection of primary and secondary outcome, core outcomes sets need to first carefully review if their included outcomes are surrogates, and if so, focus on the inclusion of validated surrogate endpoints.

### Patients and the public

A key challenge for patients and the public is their understanding of what surrogates mean and the related risks of being misled about their treatment effects and whether these translate into real benefits in terms of target outcome—be that the judgment around the relative benefit and harm of new treatment at an individual or population level. The last decade has seen a sea-change in the recognition of the importance of patient and public involvement and engagement (PPIE) in the process of research.^52^ Formal PPIE representation on the trial management and related oversight committees is now common practice in many trials and requirement of research funders. A key benefit is the opportunity to include patients and the public alongside clinicians and researchers in deciding aspects of the trial design including the primary outcome and trade off in the choice of surrogate endpoint versus final outcome. As well as involvement in individual trials, the development of recently published SPIRIT-Surrogate and CONSORT-Surrogate reporting extensions included PPIE partners, alongside trialists, clinicians, and journal editors. A SPIRIT-Surrogate extension patient-facing reporting item for protocols is to: “state whether and how trial participants will be engaged and informed before enrolment that the trial was designed to evaluate an intervention’s effect using a surrogate endpoint”.^49^

## Conclusion

The appropriate use of validated surrogate endpoints can significantly reduce clinical trial costs and accelerate patient access to new therapies. Despite widespread use by regulators, HTA agencies and payers remain cautious in adopting surrogates for reimbursement decisions. Scientific validation, demonstrated by strong treatment effect associations, is essential for the acceptance of surrogates in clinical practice. GFR slope in CKD is a ‘first in class’ exemplar of a validated surrogate. The review highlights how stakeholders can address challenges and promote the effective use of surrogate endpoints in future healthcare policymaking.

## Contributors

RST conceptualised this review article. RST, HH, LI were involved in writing and reviewing drafts; MB, OC, BD, DG, JCJM, SS, MT-B were involved in the review and editing of the drafts; all authors read and approved the final version of the manuscript and were involved in the decision to submit. RST was responsible for the decision to submit the manuscript.

## Declaration of interests

RST reports research grant from UK Medical Research Council (MRC) funding for the SPIRIT/CONSORT-Surrogate extension development (grant reference MR/V038400/1).

HJLH reports consultancy for Alexion, AstraZeneca, Alnylum, Amgen, Bayer, Boehringer Ingelheim, Dimerix, Eli-Lilly, Janssen R&D, Novartis, NovoNordisk, Roche, and Travere Therapeutics (payments to employer). He has received research support from AstraZeneca, Boehringer Ingelheim, Bayer, Janssen and NovoNordisk (payments to employer).

MB reports limited honoraria for occasional advisory roles (AstraZeneca, Boehringer-Ingelheim) and in-company teaching (Novartis), as well as research support from AstraZeneca, always ensuring the absence of conflicts of interest.

OC reports research grant from UK Medical Research Council (MRC) funding for the SPIRIT/CONSORT-Surrogate extension development (grant reference MR/V038400/1).

BD reports he has received over the last 5 years personal compensation for board participation, speaking fees and research support from Abbott, MSD, Cemka, Cerba Research, Novo-Nordisk, Sanofi Winthrop Industry, Eli Lilly, Janssen, Astra Zeneca and Continuum + company.

DG reports no conflicts of interest.

JCJ reports receiving fees for his participation as a speaker or member of advisory boards for the companies Astellas, AstraZeneca, Baxter, Bayer, Boehringer-Ingelheim, GSK, CSL Vifor, Pfizer, Pierre Fabre, Travere. Similarly, Federación Nacional de Asociaciones ALCER has received grants for projects under his direction from the companies Astellas, AstraZeneca, Baxter, BMS, Boehringer-Ingelheim, CSL, Vifor, Fresenius Medical Care, GSK, Hansa Biopharma, Ipsen Pharma, MSD, Novartis, Novo Nordisk, Palex, and Pfizer.

SS reports honoraria for advisory boards/speaker fees from AstraZeneca, Bayer, Boehringer-Ingelheim, CSL Vifor, Chiesi, Menarini, NovoNordisk, Novartis, GSK, Sobi Purespring, Inozyme, Amicus, Santhera, Stada; research support from AstraZeneca, Amgen, Novartis, CSL Vifor, J&J and Sanofi-Genzyme.

MTB reports limited honoraria for occasional advisory roles (AstraZeneca, Boehringer-Ingelheim) and in-company teaching (Novartis), as well as research support from AstraZeneca, always ensuring the absence of conflicts of interest.

LAI reports research grants from the NIH and NKF. She is a consultant for Alexion, AstraZeneca, Dimerix (payments to employer). Novartis, Novo Nordisk, Roche, and Travere Therapeutics (payments to employer). She has received research support from Alexion, Chinnocks, Novartis, Otsuka (payments to employer).

## Appendix A Supplementary data

Supplementary eFig. 1 and Tables 1–3
