# Supplementary material for: Use of surrogate endpoints in health technology assessment and reimbursement of treatments for the management of chronic kidney disease
Source: eClinicalMedicine. 2025 Aug 29;88:103465. doi: 10.1016/j.eclinm.2025.103465 (PMC12418889; doi:10.1016/j.eclinm.2025.103465)
Supplement: Supplementary eFig. 1 and Tables 1–3 [file mmc1.docx]

**e-Supplement**

**eTable 1. Summary of HTA agencies considerations for surrogate endpoints***

|  | **Definition of relevant outcomes for decision making** | **Grounds for acceptance of surrogate endpoint** | **Requirement of validation of surrogate endpoint** | **Decision making considerations of surrogate endpoint** |
| --- | --- | --- | --- | --- |
| France  HAS | The primary outcome measure of a study must be a relevant clinical endpoint wherever it is possible to collect one | Surrogate endpoint accepted if a link with a clinical morbidity and mortality endpoint has been demonstrated in the disease concerned. If a relevant clinical endpoint is not used in the trials, justification explaining this choice is expected. | No specific statement | Validated surrogate endpoints are considered as relevant as clinical endpoints when not using directly the clinical endpoint is justified. |
| Belgium  KCE | Outcomes must always be patient important. | Surrogate endpoints accepted only when high-quality evidence regarding important outcomes is lacking. | No specific statement | The necessity to substitute with the surrogate may ultimately lead to rating down the quality of the evidence because of indirectness. |
| Italy  AIFA | Preference for outcomes recognised as clinically relevant and validated for  the pathology in question. It is requested to provide information useful for assessing the impact of the condition in terms of mortality, disability, years of life lost due to disability and premature death (DALYs), quality of life of patients and any aspect characterising the impact of the clinical condition on patients and  on society. | No specific statement. | No specific statement. | The impact of the Product in terms of health outcomes must be determined on the basis of expected change in mortality and/or other health events (slowing of disease progression and/or reduction of severity) in comparison with the alternatives. Scenario analyses examining a spectrum of possible circumstances are especially required when efficacy data are based on surrogate endpoints with an uncertain effect on final outcomes. |
| UK  NICE | Relevant outcomes include any health outcomes resulting directly or indirectly from any technologies being evaluated. | The use of a surrogate endpoint is acceptable, with 3 levels of evidence:  •Level 3: Biological plausibility of relation between surrogate endpoint and final outcomes.  •Level 2: Consistent association between surrogate endpoint and final outcomes derived from epidemiological or observational studies.  •Level 1: The technology's effect on the surrogate endpoint corresponds to commensurate effect on the final outcome as shown in the RCT. | Preferably comes from a meta-analysis of level 1 evidence (that is, RCTs) that reported both the surrogate and the final outcomes, using the recommended meta-analytic methods. | Uncertainty associated with the relationship between the surrogate end points and the final outcomes should be quantified and presented. It should also be included through probabilistic sensitivity analysis and can be further explored in scenario analysis. |
| Spain  AEMPS | Accepts surrogate endpoints for measuring the clinical benefit of a drug under certain circumstances** | These endpoints are particularly used when measuring the final clinical benefit, such as overall survival, would be impractical or would take too long. Surrogate endpoints must be scientifically validated to ensure they provide an accurate indication of the actual clinical benefit expected from the treatment. | No specific statement. | No specific statement. |
| Germany  IQwiG/G-BA | Patient benefit assessed by increase in life expectancy, improvement in health status and quality of life, and reduction in disease duration and adverse effect. | Surrogate endpoints only considered if validated using appropriate statistical methods within a sufficiently restricted patient population and within comparable intervention. | Correlation-based validation is the preferred method and surrogate threshold effect. If validation study shows statistically low correlation (R ≤ 0.7; R^2^ ≤ 0.49) measured at the lower bound of the confidence interval then the surrogate is not considered as a valid endpoint. | Assessment of surrogate endpoints lead to a reduction in the certainty of conclusions. |
| Europe  EU-HTA | Preference for long-term or final outcomes (i.e., the occurrence of an irreversible event of primary interest such as death) are preference. | Surrogate endpoint provides an indirect measurement of effect in situations in which direct measurement of a patient-centred effect is not feasible or practical. Surrogate endpoints should be adequately validated: the surrogate-final endpoint relationship must have been demonstrated based on biological plausibility and empirical evidence.  It should be explained for which outcome of interest surrogacy is claimed and the demonstration of the strength of the association between the surrogate outcome and the outcome of interest and treatments effects should be provided. | Correlation from meta-analyses of several RCTs between the effects on the surrogate and the effects on the clinical endpoint – consider [strong correlation] values of 0.85 and 0.95. If there is no high correlation demonstrated, conclusions might still be made if the surrogate threshold effect is considered. | No specific statement. |

^1^AIFA awards 5-levels of added therapeutic value possible - Maximum, Important, Moderate, Poor, Absent – for the recognition of innovativeness status

AEMPS: Agencia Española de Medicamentos y Productos Sanitarios [Spanish Agency of Medicines and Medical Devices]; AIFA: Agenzia Italiana del Farmaco [Italian Medicines Agency]; HAS: Haute Autorité de Santé [French National Authority for Health]; G-BA: Gemeinsamer Bundesausschuss [German Federal Committee]; IQWiG: Institut für Qualität und Wirtschaftlichkeit im Gesundheitswesen [Institute for Quality and Efficiency in Health Care]; KCE: Belgian Health Care Knowledge Centre; NICE: National Institute for Health & Care Excellence;

*Based on analysis of details available of HTA agencies websites.

**In Spain, the definition of relevant outcomes for the assessment and approval of medications and medical technologies, generally follows national guidelines (AEMPS) but can indeed vary at a subnational level, including Autonomous Communities (CCAA) and institutional/hospital formularies

## **eTable 2. Trial-level results using secondary endpoint i.e. dialysis or GFR <15 ml/min per 1.73 m^2^**

|  | Event | Meta-regression slope  (95% BCI) | Intercept  (95% BCI) | R^2^ trial  (95% BCI) |
| --- | --- | --- | --- | --- |
| Total slope computed at 3 years | KFRT, doubling of serum creatinine or GFR < 15 | -0.35 (-0.42 to -0.29) | -0.04 (-0.09 to 0.01) | 0.97 (0.82 to 1.00) |
|  | KFRT or GFR < 15 | -0.22 (-0.31 to -0.12) | -0.10 (-0.17 to -0.04) | 0.92 (0.56 to 0.99) |
| Chronic slope | KFRT, doubling of serum creatinine or GFR < 15 | -0.33 (-0.46 to -0.20) | -0.01 (-0.10 to 0.10) | 0.55 (0.25 to 0.77) |
|  | KFRT or GFR < 15 | -0.27 (-0.33 to -0.21) | -0.11 (-0.17 to -0.06) | 0.87 (0.64 to 0.97) |

KFRT: composite of kidney failure with replacement therapy (initiation of chronic treatment with dialysis or kidney transplantation)

BCI: Bayesian confidence interval

**eTable 3. HTA Case study - SGLT-2 inhibitors for the treatment of chronic kidney disease**

|  | **Empagliflozin for CKD** | | **Dapagliflozin for CKD** | |
| --- | --- | --- | --- | --- |
|  | **Coverage decision** | **Consideration of GFR slope/evidence** | **Coverage decision** | **Consideration of GFR slope/evidence** |
| **NICE (England, Wales, Northern Ireland)** | Recommended (restricted)* [TA942] | No specific consideration of eGFR slope evidence other than to define the population in scope of the appraisal as per clinical trial population. | Recommended (restricted)** [TA775] | No specific consideration of eGFR slope evidence other than to define the population in scope of the appraisal as per clinical trial population. |
| **SMC (Scotland)** | Recommended with restriction* [SMC2642] | “The primary outcome was mainly driven by an improvement in the number of patients having an eGFR reduction ≥40%, however every component of the composite outcome had a lower number of events in the empagliflozin group versus placebo” | Accepted with restriction* [2763] | “DAPA-advKD was a randomised, open label study comparing dapagliflozin plus integrated CKD care with integrated CKD care only in patients with CKD stages 4 and 5 (eGFR ≥ 10 to < 30 mL/min/1.73m2).6 There was no restriction on uACR level. Patients randomised to receive dapagliflozin had improvements in the key outcome of eGFR slope as well as renal and cardiovascular outcomes compared with integrated CKD care alone.” |
| **IQWiG/G-BA, Germany** | Reimbursed  Added benefit not proven | Trial comparator (SoC, but SGLT2i not allowed or insufficiently used) deemed not appropriate (Dapagliflozin should be part of SoC).***  Total and chronic eGFR slope presented complementary in value dossier (without surrogate validation), but study not assessed by G-BA due to inadequate comparator. | Reimbursed  CKD without symptomatic, chronic HF: Hint for considerable added benefit (based on benefits in all-cause mortality, ESKD, all-cause hospitalization and SAE)  CKD with symptomatic chronic HF: Hint for minor added benefit (based on benefits in all-cause hospitalization and SAE) | Total eGFR slope presented complementary in value dossier (without surrogate validation) but not assessed by G-BA. |
| **HAS, France** | Reimbursed  Notes minor clinical benefit (CAV IV) | Notes that treatment effect driven by decrease in GFR and lack of HRQoL data | Reimbursed  Notes minor clinical benefit (CAV III) | Notes absence of HRQoL data & consideration of current treatment alternatives |
| **Spain**  **AEMPS** | Reimbursed | “The EMPA-KIDNEY study has shown benefit over placebo in slowing renal progression in patients with CKD (eGFR ≥20 and <45 ml/min/1.73m^2^; or eGFR between 45 and <90 ml/min/1.73m^2^ and a CACo≥200 mg/g), whether or not they had diabetes.” | Reimbursed | “Dapagliflozin has been shown to be effective and safe in the treatment of patients with CKD with eGFR ≥25 and ≤75 ml/min/1.73m2 and ACR ≥200 mg/g with or without T2DM, added to optimized standard treatment with iRAS.” |
| **Italy**  **AIFA** | Reimbursed (Class A) | Not stated | Reimbursed (Class A) | Not stated |

*More restricted population than licence to reflect trial inclusion criteria.

*More restricted population than licence to reflect trial inclusion criteria: dapagliflozin is recommended as an option for treating CKD in adults. It is recommended only if: it is an add-on to optimised standard care including the highest tolerated licensed dose of angiotensin-converting enzyme (ACE) inhibitors or angiotensin-receptor blockers (ARBs), unless these are contraindicated, and people have an eGFR of 25 ml/min/1.73 m^2^ to 75 ml/min/1.73 m^2^ at the start of treatment and: have type 2 diabetes or have a uACR of 22.6 mg/mmol or more.

***For empagliflozin, there was no added benefit as dapagliflozin+ renin–angiotensin–aldosterone system inhibitors (RAASi) became standard of care by the time of empagliflozin submission to G-BA, hence, EMPA-KIDNEY comparator arm (placebo on top of standard cafre of RAASi) was deemed as not relevant for Germany.

AIFA: Agenzia Italiana del Farmaco [Italian Medicines Agency]; AEMPS: Agencia Española de Medicamentos y Productos Sanitarios [Spanish Agency of Medicines and Medical Devices]; HAS: Haute Autorité de Santé [French National Authority for Health]; G-BA: Gemeinsamer Bundesausschuss [German Federal Committee]; IQWiG: Institut für Qualität und Wirtschaftlichkeit im Gesundheitswesen [Institute for Quality and Efficiency in Health Care]; NICE: National Institute for Health & Care Excellence; SMC: Scottish Medicines Consortium; HRQoL: health-related quality of life.

**eFigure 1. Renal nerve denervation for management of refractory hypertension – example of HTA handling of surrogate endpoint (SBP) [from 54]**

**Reference:**

Taylor R.S., Bentley A., Metcalfe K., et al. Cost effectiveness of endovascular ultrasound renal denervation in patients with resistant hypertension. Pharmacoecon Open. 2024;8:525–537.
